# Retraction Notice to: Perceptual load affects spatial tuning of neuronal populations in human early visual cortex

**DOI:** 10.1016/j.cub.2020.11.015

**Published:** 2020-12-07

**Authors:** Benjamin de Haas, D. Samuel Schwarzkopf, Elaine J. Anderson, Geraint Rees

(Current Biology *24*, R66–R67; January 20, 2014)

In our paper, we reported a significant increase of parafoveal population receptive field (pRF) sizes and eccentricity in visual areas V1–3 under high versus low perceptual load at fixation. We have recently been notified of a potential flaw in our analysis pipeline for this paper. As described in the original manuscript, the analysis defined eccentricity bands according to one of the two conditions that were then compared (the low-load condition). This is currently an approach that is widespread in the field, but we now realize has an unappreciated potential bias. The circularity can bias the results due to a combination of regression to the mean and heteroskedastic error variance (eccentricity errors are larger in the periphery than in the central visual field). Also, as reported, the original analysis expressed changes in pRF sizes proportionally. This can curtail the negative end of the difference distribution because growth, but not shrinkage, can be greater than 100%. Simulations and re-analyses of our original data strongly suggest that these steps did indeed inflate the effects we reported. Specifically, we conducted a re-analysis using absolute rather than proportional changes in pRF sizes, and binning data according to independent probabilistic maps. Reassuringly, this analysis reproduced trends for increased pRF sizes in V1–3 under high versus low load. However, these no longer survived family-wise error correction (FWE). Likewise, a trend for the reported increase in pRF eccentricity was now only observed in V1 and failed to reach FWE significance.

Therefore, we no longer consider the reported results reliable and wish to correct the scientific record by voluntarily retracting our paper. We apologize to the scientific community for any inconvenience caused and caution fellow researchers against the use of non-independent binning practices, which appear widespread in the field. Finally, we would like to thank our colleague Susanne Stoll, who first pointed out the problem to us and plans to publish in due course a more general exposition on the difficulties of this approach.

